# Treatment of Unstable Occipital Condylar Fractures in Children—A STROBE-Compliant Investigation

**DOI:** 10.3390/medicina57060530

**Published:** 2021-05-25

**Authors:** Ryszard Tomaszewski, Artur Gap, Magdalena Lucyga, Erich Rutz, Johannes M. Mayr

**Affiliations:** 1 Department of Pediatric Traumatology and Orthopedics, Upper Silesian Children’s Health Centre, Medyków 16, 40-752 Katowice, Poland; tomaszewskir@gmail.com (R.T.); a.gap1@wp.pl (A.G.); magdalenalucyga@gmail.com (M.L.); 2 Institute of Biomedical Engineering, Faculty of Science and Technology, University of Silesia, Medyków 16, 40-752 Katowice, Poland; 3 Department of Orthopaedics, The Royal Children’s Hospital Melbourne, Melbourne, VIC 3052, Australia; erich_rutz@hotmail.com; 4 Department of Pediatric Surgery, University Children’s Hospital Basel, University of Basel, 4031 Basel, Switzerland

**Keywords:** occipital condyle fracture, children, halo vest, CT, MRI

## Abstract

*Background and objectives:* Occipital condyle fractures (OCF) occur rarely in children. The choice of treatment is based on the Anderson–Montesano and Tuli classification systems. We evaluated the outcome of unstable OCF in children and adolescents after halo-vest therapy. *Materials and Methods:* We treated 6 pediatric patients for OCF, including 3 patients (2 girls, 1 boy) with unstable OCF. Among the 3 patients with unstable OCF, 2 patients presented with an Anderson–Montesano type III and Tuli type IIB injury, while 1 patient had an Anderson–Montesano type I fracture (Tuli type IIB) accompanied by a C1 fracture. On admission, the children underwent computed tomography (CT) of the head and cervical spine as well as magnetic resonance imaging (MRI) of the cervical spine. We treated the children diagnosed with unstable OCF with halo-vest immobilization. Before removing the halo vest at the end of therapy, we applied the CT and MRI to confirm OCF consolidation. At follow-up, we rated functionality of the craniocervical junction (CCJ) based on the Neck Disability Index (NDI) and Questionnaire Short Form 36 Health Survey (SF-36). *Results*: All children achieved OCF consolidation after halo-vest therapy for a median of 13.0 weeks (range: 12.5–14.0 weeks). CT and MRI at the end of halo-vest therapy showed no signs of C0/C1 subluxation and confirmed the correct consolidation of OCF. The only complication associated with halo-vest therapy was a superficial infection caused by a halo-vest pin. At follow-up, all children exhibited favorable functionality of the CCJ as documented by the NDI score (median: 3 points; range: 3–11 points) and SF-36 score (median: 91 points; range: 64–96 points). *Conclusions*: In our small case series, halo-vest therapy resulted in good mid-term outcome in terms of OCF consolidation and CCJ functionality. In pediatric patients with suspected cervical spine injuries, we recommend CT and MRI of the CCJ to establish the diagnosis of OCF and confirm stable fracture consolidation before removing the halo vest.

## 1. Introduction

In children, occipital condyle fractures (OCF) represent a rare injury of the craniocervical junction (CCJ), occurring in 1% to 3% of cervical spine injuries [1,2]. This injury is most prevalent in patients suffering from polytrauma, especially those with head injuries [1,3,4,5]. In most of these patients, computed tomography (CT) of the head and cervical spine is performed on hospital admission, which facilitates early diagnosis of OCF [6,7]. The most common OCF classification systems, i.e., Anderson–Montesano [8] and Tuli [9] classifications, are based on CT and magnetic resonance imaging (MRI) findings and allow us to assess CCJ stability.

While treatment of stable OCF relies on conservative management using cervical braces to immobilize the CCJ [10], unstable OCF requires halo-vest immobilization or surgical stabilization, particularly if complicated by neurologic deficits [1,4,11,12,13]. However, the literature on OCF treatment in children and adolescents is limited, consisting mainly of individual case reports. We evaluated the outcome of unstable OCF in a small series of children and adolescents treated with halo-vest immobilization.

## 2. Materials and Methods

We treated 6 patients with OCF between 2004 and 2018. Three of these children (2 girls, 1 boy) were diagnosed with unstable OCF. The median age of the 3 children was 15.2 years (range: 15–18 years). In all 3 cases, OCF was caused by road traffic incidents and was accompanied by other severe injuries (Table 1). After admission to the accident & emergency (A&E) room, the children underwent CT of the head and cervical spine as well as MRI of the cervical spine. We refrained from obtaining plain X-ray images of the cervical spine.

Among the 3 patients with unstable OCF, 2 patients had Anderson–Montesano type III [8] and Tuli type IIB [9] injuries, while 1 patient presented with an Anderson–Montesano type I fracture (Tuli type IIB injury) accompanied by a C1 fracture. None of our patients had bilateral OCF. The 3 patients with unstable OCF started halo-vest therapy at a median of 6 h (range: 3–18 h) after hospital admission. To confirm correct fracture alignment, we obtained a control CT at a median of 1.3 days (range: 1.0–2.0 days) after initiating halo-vest therapy. Before removing the halo vest at the end of treatment (median duration: 13.0 weeks; range: 12.5–14.0 weeks), we confirmed fracture consolidation by means of a CT of the CCJ. At a median of 5.6 days (range: 3.0–9.0 days) after removing the halo vest, all patients underwent an MRI of the cervical spine.

The patients were followed up for a median of 49 months (range: 6–72 months). At the follow-up assessment, we evaluated CCJ functionality after obtaining approval by the Ethics Committee of the Upper Silesian Children’s Health Centre, Katowice, Poland. We rated CCJ functionality by means of the Neck Disability Index (NDI) and Questionnaire Short Form 36 Health Survey (SF-36) [14,15]. To adjust the questionnaires to the young age of our patients, we excluded point 8 (“driving a car”) in the NDI and exchanged the wording of questions 4, 5, and 8 (“problems at work”) with “problems at school” in the SF-36.

## 3. Results

Table 1 shows patient characteristics, Anderson–Montesano [8] and Tuli [9] fracture classifications, cause of injury, accompanying injuries, and immobilization method for all OCF patients (*n* = 6).

In 2 patients (P.P., K.D.) with Anderson–Montesano type III and Tuli type IIB injuries, CT and MRI obtained after the injury revealed a translation of 3 mm and 4 mm between C0 and C1 and rotational displacement of C0/C1 by 10 and 12 degrees, respectively. Moreover, MRI confirmed intact ligaments, especially the alar ligament, apical ligament, and tectorial membrane.

**Figure 1:**
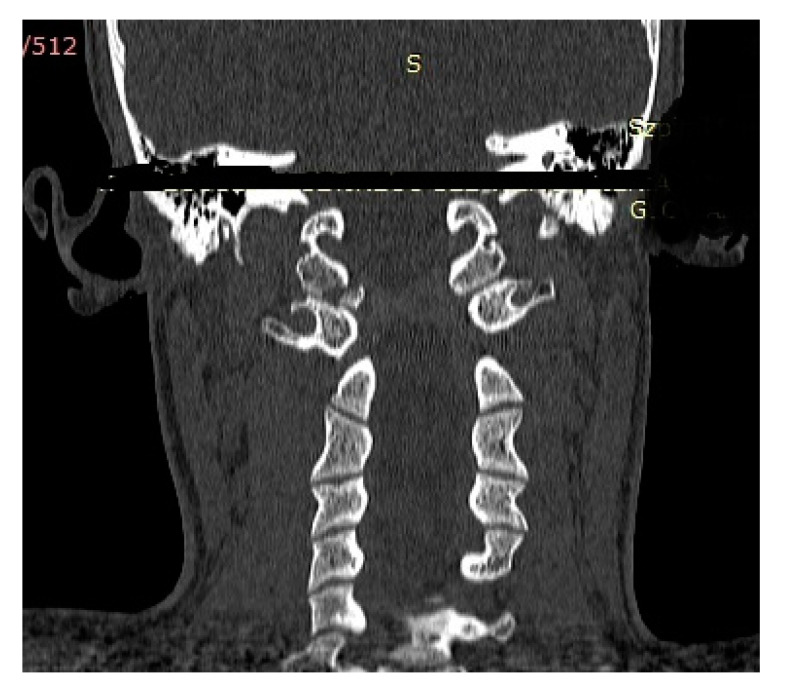
CT scan (frontal plane) of a boy aged 15.2 years (Anderson–Montesano type III, Tuli type IIB injury; patient: P.P.) obtained after admission to the A&E room. Avulsed fragment of right condyle displaced medially by 4.0 mm.

**Figure 2:**
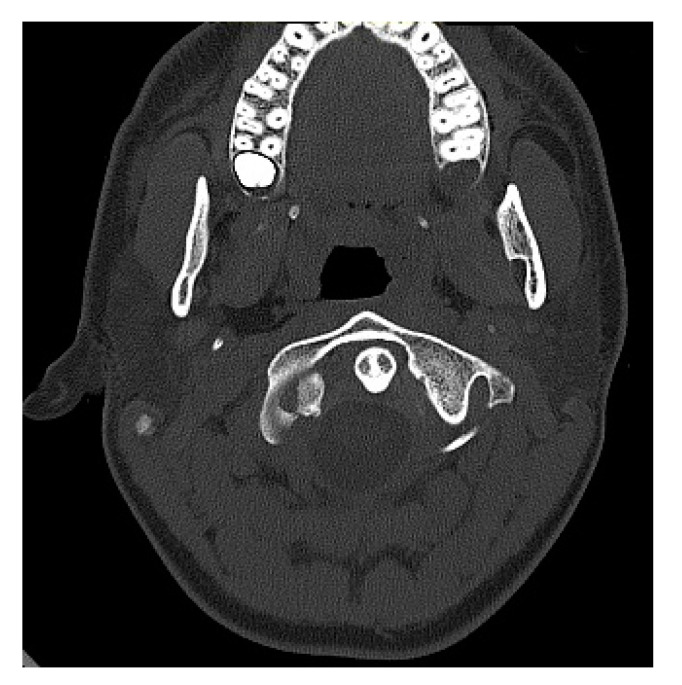
CT scan (transverse plane) of a boy aged 15.2 years (patient: P.P.) obtained after admission to the A&E room. Avulsed fragment of right condyle displaced medially by 4.0 mm. Dislocation of odontoid to the left.

Figure 1 and Figure 2 show the CT findings (frontal and transverse planes) for one of these patients (patient P.P.) after admission to the A&E room.

**Figure 3:**
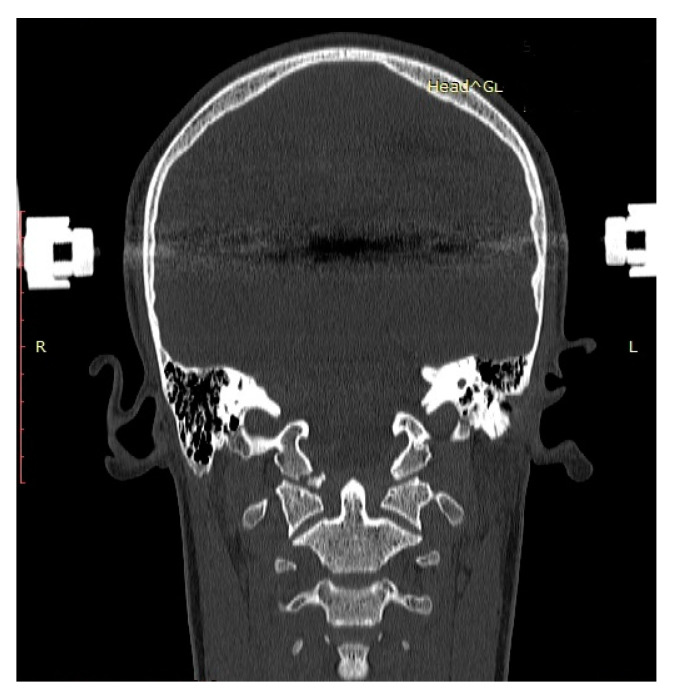
Control CT scan (frontal plane) of a boy aged 15.2 years (patient: P.P.) obtained after initiating halo-vest therapy and closed reduction of right-sided OCF. Correct alignment of condyle fragment on the right side and diminished fracture gap.

The control CT obtained after initiating halo-vest therapy to reduce and stabilize OCF showed a displacement (1 mm) by separation of C1 from the occipital condyle and C0/C1 translation by 1 mm, but no malrotation (rotational difference of C0 and C1: 0 degrees; Figure 3).

### 3.1. Outcome of Unstable OCF After Halo-Vest Therapy

**Figure 4:**
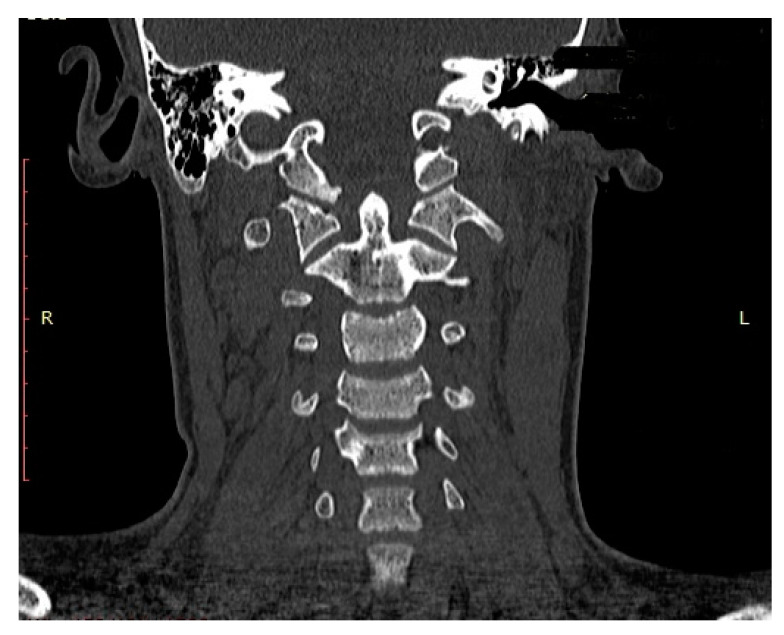
CT scan (frontal plane) of a boy aged 15.2 years (patient: P.P.) obtained after halo-vest therapy, showing bony consolidation of right-sided OCF in correct alignment.

**Figure 5:**
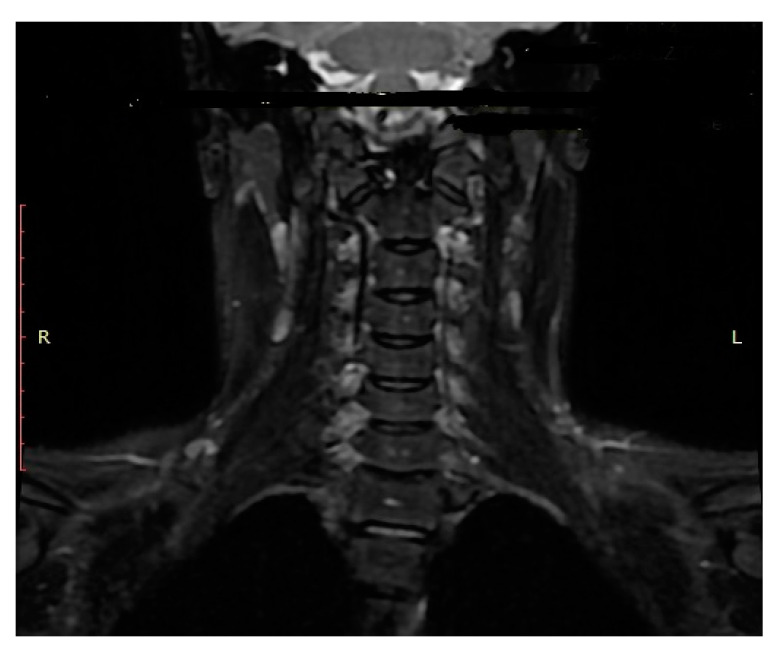
MRI scan (frontal plane) of a boy aged 15.2 years (patient: P.P.) confirming union of right-sided OCF in correct alignment.

Halo-vest therapy in our patients with unstable OCF lasted for a median of 13.0 weeks (range: 12.5 to 14.0 weeks). CT and MRI obtained at the end of the halo-vest therapy showed no signs of C0/C1 subluxation in the C0/C1 joint and confirmed correct consolidation of OCF in all patients. Figure 4 and Figure 5 show the CT and MRI scans for patient P.P.

### 3.2. Functionality of Craniocervical Junction at Follow-up

Our patients were followed up for a median period of 49 months (range: 6–72 months). With respect to CCJ functionality determined at follow-up, we calculated a median score of 3 points (range: 3–11 points) using the modified NDI, while the SF-36 classification yielded a median score of 91 points (range: 64–96 points; Table 2).

### 3.3. Complications of Halo-Vest Therapy

Halo-vest therapy was well tolerated in all children. The only complication was a superficial infection caused by one of the halo-vest pins, which occurred on the 45th day after treatment start (patient R.M.). This infectious complication (Clavien-Dindo type III complication, [16]) did not require any antibiotic therapy, but the position of the pin had to be changed.

## 4. Discussion

The main findings of this study in pediatric patients were the confirmation of suspected OCF based on CT of the CCJ and the need to diagnose accompanying ligament or nerve injuries using MRI. MRI assesses the spinal cord and helps to diagnose possible vascular injuries. In our series of pediatric patients with unstable OCF, the halo-vest was used with a good mid-term outcome.

OCF occurs in 1% to 3% of cervical spine injuries [8,17], and the associated mortality rate is high (8.3%–16.0%) [9]. OCF has frequently been detected in post-mortem examinations, including the first report of OCF by Bell in 1817 [1,8,17,18]. OCF occurs mainly in adolescents and young male adults [19], which agrees with our observation. The median age of our patients with unstable OCF was 15.2 years (range: 15–18 years). To the best of our knowledge, the youngest patient reported in the literature was aged 7 months at the time of injury [20]. Moreover, OCF before adolescence has mostly been reported in case reports [13,20,21].

OCF is often accompanied by polytrauma, and the most common causes of condylar fractures are high-energy injuries, especially road traffic incidents [1,2,22]. Our 3 patients suffering from unstable OCF were all injured in traffic incidents. Nonetheless, such fractures in children may occasionally be caused by low-energy injuries. For example, Kapapa et al. presented a case of OCF in a 15-year-old child who fell from low height [2].

In as many as 75% of adult cases, OCF is accompanied by Collet-Sicard syndrome, which is characterized by paralysis of the lower four cranial nerves because these nerves are close to the site of OCF [1,3,4,5]. Moreover, damage to the vertebral artery may accompany OCF, as pointed out by Burks et al. [5]. In our patients, we did not observe any injury to peripheral nerves or vertebral artery. Our observation agrees well with literature reports, confirming that paralysis of the lower four cranial nerves (Collet-Sicard syndrome) and lesions of the vertebral artery are extremely rare in children and adolescents. Therefore, such symptoms are not reliable for the diagnosis of OCF fractures in children.

Radiologic diagnosis of OCF based only on plain X-rays is difficult and unreliable [23]. In plain X-ray images (AP and lateral views), the superimposing structure of the mandible and occipital bone interfere with the occipital-condylar image. In some cases, prevertebral soft-tissue edema is visible in the lateral view [24], but the role of this sign in plain X-ray images is limited. On the other hand, open-mouth radiography can help to diagnose condylar injuries by partially exposing the occipital condyle [11,24,25].

Nonetheless, the method of choice in terms of radiologic diagnostics is CT of the CCJ [6,7,12,25,26,27], which provides excellent imaging of bone structures of the CCJ. In all our patients, we performed CT scans of the head and cervical spine immediately after admission to the A&E room without obtaining plain X-ray images. We assessed the quality of fracture reduction after halo-vest immobilization, bone consolidation at the end of treatment, as well as C0/C1 instability on the basis of CT and MRI scans. For identifying cervical spine injuries, combined CT and MRI examinations proved more reliable than CT combined with plain X-ray images [28]. MRI is particularly useful in the assessment of alar and transverse ligaments as well as capsular injures, especially in the T2 view [17]. Mueller et al. proposed exclusive use of MRI in children to avoid unnecessary exposure to ionizing radiation associated with CT [17]. However, to the best of our knowledge, there are no literature data on emergency imaging of OCF fractures by MRI. Our MRI scans allowed us to evaluate the CCJ and assess ligament status, which is considered crucial in evaluating fracture stability [29].

Few systems to classify OCF in children have been proposed, probably because this jury is rare in children. Thus, there is a lack of reliable criteria to assess OCF stability, especially in pediatric patients. The Anderson–Montesano classification established in 1988 is still commonly used [8]. The authors divided OCF into 3 types, based on the mechanism of injury as described below.

### 4.1. Anderson–Montesano Classification

Type I injury is caused by compression, and OCF is frequently characterized by a fracture with minimal displacement accompanied by possible damage to the alar ligament [24]. However, type I fractures are considered stable.

Anderson–Montesano type II injury is caused by a direct blow. Additionally, this injury type includes a fracture of the skull base. Because the ligaments remain uninjured, this type of OCF represents a stable fracture [24].

Type III fracture is caused by lateral/rotational bending with ligament damage rendering it potentially unstable. Although the Anderson–Montesano classification is easy to apply in general, it is difficult to decide between conservative and surgical treatment in Anderson–Montesano type III OCF. Notably, Anderson–Montesano treated a type III OCF injury non-operatively using a rigid cervical orthosis [9]. Hanson et al. emphasized the difficulty of identifying stable type I and III fractures according to Anderson–Montesano and of selecting those OCF types that should undergo operative stabilization [24]. We agree with Hanson et al. [24] that the distinction between type I and type III injuries may be difficult.

### 4.2. Other Classifications

In 1997, Tuli et al. presented an OCF classification system based on the stability of the C0/C2 joint complex [9]. They divided OCF into type I, type IIA, and type IIB injuries. Here, type I injury is characterized by an undisplaced, stable C0/C2 joint, while OCF type IIA represents displacement of the complex OCF with preserved C0/C2 stability. Type IIB is defined as displaced OCF with an unstable C0/C2 complex. Tuli et al. defined OCF instability in the C0/C2 complex in terms of rotation by >8° and translation >1 mm at the level of C0/C1, in combination with a translation C1/C2 of >4 mm, overhang of C1 on C2 by >7 mm, axial rotation >45°, and C1/C2 translation with avulsion of the transverse ligament. In contrast, Hanson et al. proposed a stability criterion based on CT scans in adult patients with unilateral OCF with contralateral widening of the atlanto-occipital distance of >2 mm [24].

In our series of pediatric patients, 1 patient required halo-vest treatment due to OCF and C1 fracture. CT and MRI of the remaining 2 patients with unstable fractures revealed rotational displacements of 10° and 12° at the level of C0/C1, coupled with translation of 4 mm and 3 mm at C0/C1, respectively.

In 2012, Mueller et al. proposed an OCF classification system encompassing unilateral as well as bilateral injuries, which they observed in 4 of 31 patients [17]. The authors did not propose variable treatment strategies for unilateral or bilateral OCF, but reported higher mortality in patients with bilateral OCF. The classification proposed by Mueller et al. consists of 3 categories, namely stable fractures type 1 (unilateral OCF without atlanto-occipital dislocation) and type 2 (bilateral OCF without atlanto-occipital dislocation), as well as unstable type 3 unilateral or bilateral OCF with atlanto-occipital dislocation. The authors did not define the criteria for assessing atlanto-occipital subluxation [17].

### 4.3. Patient Management

We observed two Anderson–Montesano type III fractures with avulsion fracture of the alar ligament and displacement of 3 mm and 4 mm at the level of C0/C1, respectively. MRI confirmed intact C0/C2 ligaments. We managed these pediatric patients successfully with halo-vest immobilization. Therefore, we hypothesize that C0/C1 subluxation >1 mm and rotation >8° are a reliable indication for halo-vest therapy, especially in pediatric patients [8,25]. It is worth noting that in children below the age of 8 to 9 years, the majority of cervical spine fractures involve the C1/C3 region, mostly because of large amounts of growth cartilage and vulnerable, growing bone in the upper cervical spine. Therefore, the incidence of OCF in this age group is extremely low [13,20,24]. OCF occurs more frequently in patients during or after puberty [1,3,30], which agrees with the age range of our pediatric patients.

### 4.4. Conservative Treatment Options for OCF

Conservative treatment options for OCF involve the use of rigid collar orthoses and Minerva braces. Orthoses and braces are applied in stable fractures, i.e., Anderson–Montesano types I, II, and rarely type III injuries and Tuli types I and IIA injuries [4,23,31]. Notably, Anderson–Montesano type III injuries occur in approximately 75% of patients suffering from OCF [24]. In all these patients, a decision between conservative or surgical treatment has to be taken. We agree with other authors that the most important criterion for decision making regarding the type of treatment is accurate assessment of fracture stability [1,4,18,32].

Halo-vest immobilization or internal fixation is recommended for unstable fractures [1,9,12,13]. Malham et al. recommended halo-vest immobilization for approximately 6 to 12 weeks in patients suffering from unstable OCF without compression on the spinal cord at the level of the CCJ [4]. We applied halo-vest immobilization for approximately 13 weeks in our pediatric patients.

### 4.5. Operative Stabilization of OCF

Open reduction and internal fixation (ORIF) with occipital cervical fusion is indicated in patients with neurologic deficits [11]. Maserati et al. mentioned the possibility of late surgical stabilization in patients initially treated non-operatively with cervical orthoses [12]. Karam & Traynalis emphasized the role of immobilization to achieve bone consolidation and recover peripheral nerve function [31].

In line with Shin et al. [32], we observed no complications associated with halo-vest immobilization and obtained stable consolidation of OCF in all 3 patients. Therefore, we propose halo-vest immobilization as first-line treatment in pediatric patients with OCF. In addition, we encourage the development of a new OCF classification system to improve assessment as well as stability prediction in children suffering from OCF. We feel that an instrument enabling accurate diagnosis and optimal treatment of OCF is urgently needed because OCF incidence rates in children and adolescents appear to be on the increase. Such a classification system should be based on prospective, randomized, multicenter studies.

### 4.6. Study Limitations and Strengths

The main limitations of our study comprise the retrospective study design and small number of patients treated for stable and unstable OCF. We evaluated fracture stability by CT and MRI of the CCJ and upper cervical spine in all our patients, but applied different OCF classification systems.

## 5. Conclusions

1. We recommend CT and MRI of the CCJ to diagnose OCF and confirm post-therapeutic fracture consolidation in pediatric patients.
2. Halo-vest immobilization in pediatric patients suffering from acute, unstable OCF provides good treatment outcomes.
3. Prospective, randomized, multicenter studies are required to confirm our findings and to establish guidelines for assessing post-traumatic stability of the CCJ in children and adolescents with OCF.

## Figures and Tables

**Table 1:** Patient characteristics, Anderson–Montesano and Tuli fracture classifications, cause of injury, accompanying injuries, and immobilization method.

Name (Initials)	Sex (Male/ Female)	Age (Years)	Anderson Montesano Classification	Tuli Classification	Cause of Injury	Accompanying Injuries	Immobilization Method
P.P.	M	15.2	III (unstable)	IIB	Road traffic accident (car passenger)	Fracture frontal bone, fracture frontal sinus, contusion of frontal lobe	Halo-vest immobilization: 12.5 weeks
K.D.	F	15	III (unstable)	IIB	Pedestrian hit by car	Lung contusion, brain concussion, multiple abrasions	Halo-vest immobilization: 13 weeks
R.M.	F	18	I (unstable)	IIB	Road traffic accident (car passenger)	Pneumothorax, neurogenic vocal cord injury, post-traumatic aphasia	Halo-vest immobilization: 14 weeks
S.D.	M	14.7	III (stable)	IIA	Road traffic accident (car passenger)	Fracture of frontal bone, fracture of nasal bone, subdural hematoma	Minerva-brace immobilization
B.W.	F	16	I (stable)	IIA	Fall from a height	Fracture of frontal bone, fracture of nasal bone, subarachnoid hemorrhage, fracture of transverse process Th3-5, fracture of radius	Minerva-brace immobilization
M.O.	M	16.1	I (stable)	IIA	Bicycle incident	Fracture frontal bone, fracture maxillary sinus, fracture orbit, metacarpal fracture	Minerva-brace immobilization

**Table 2:** NDI scores and percentages [14] and SF-36 scores [15] in patients with unstable OCF (*n* = 3) at follow-up.

Name (Initials)	NDI Scores (Percentage)	SF-36 Scores
P.P.	3/45 (6.7)	96
K.D.	3/45 (6.7)	91
R.M.	11/45 (24.4)	64

## Data Availability

The datasets generated and/or analyzed during the current study are available from the corresponding author on reasonable request.
